# Dr. Joseph S. Lehman

**Published:** 1917-04

**Authors:** 


**Dr. Joseph S. Lehman,** Buffalo 1893, died at his home in

Clarence Center, N. Y., March 14, 1917, aged 70. He had been in bad health for several years. Dr. Lehman was, at one time, a student of the editor, and we wish to express a personal regret at his death and a recognition of his noble qualities.

